# Arbutoid mycorrhizas of the genus *Cortinarius* from Costa Rica

**DOI:** 10.1007/s00572-016-0688-1

**Published:** 2016-03-11

**Authors:** K. Kühdorf, B. Münzenberger, D. Begerow, J. Gómez-Laurito, R. F. Hüttl

**Affiliations:** Leibniz Centre for Agricultural Landscape Research (ZALF), Institute for Landscape Biogeochemistry, Eberswalder Straße 84, 15374 Müncheberg, Germany; Ruhr-University of Bochum, AG Geobotany, Universitätsstraße 150, 44780 Bochum, Germany; University of Costa Rica, Escuela de Biología, San José, CP 11501-2060 Costa Rica; Brandenburg University of Technology Cottbus-Senftenberg, Chair of Soil Protection and Recultivation, Box 101344, 03013 Cottbus, Germany; German Research Centre of Geosciences Potsdam (GFZ), Telegrafenberg, 14473 Potsdam, Germany

**Keywords:** Anatomy, Central America, Morphology, Secondary cloud forest

## Abstract

Arbutoid mycorrhizas of *Comarostaphylis arbutoides* (Arbutoidea, Ericaceae) from neotropical montane forests are rarely described. To date, only mycorrhizal associations with the fungal species *Leccinum monticola*, *Leotia lubrica* and *Sebacina* sp. are known from literature. The genus *Cortinarius* is one of the most species-rich ectomycorrhizal taxa with over 2000 assumed species. In this study, two sites in the Cordillera de Talamanca of Costa Rica were sampled, where *Com. arbutoides* is endemic and grows together with *Quercus costaricensis*. Using a combined method of rDNA sequence analysis and morphotyping, 33 sampled mycorrhizal systems of *Cortinarius* were assigned to the subgenera *Dermocybe*, *Phlegmacium* and *Telamonia*. Specific plant primers were used to identify the host plant. Here, we present the phylogenetic data of all found Cortinarii and describe four of the arbutoid mycorrhizal systems morphologically and anatomically.

## Introduction

*Comarostaphylis arbutoides* is a tropical woody plant of Central America, occurring in dry oak-pine and cloud forests, as well as in the páramo at an elevation of c. 2500–3430 m a.s.l. Together with *Arbutus* and *Arctostaphylos*, it belongs to the ericaceous subfamily Arbutoidea, which are known to form arbutoid mycorrhizas with ectomycorrhizal fungi (Molina and Trappe 1982). Although, Bidartondo and Bruns (2001) infer that *Com. arbutoides* forms arbutoid mycorrhizas with diverse species of Basidiomycetes and Ascomycetes, only mycorrhizal associations with *Leccinum monticola*, *Sebacina* sp. and *Leotia* cf. *lubrica* have been described so far (Osmundson et al. 2007; Kühdorf et al. 2014, 2015). However, typical as well as presumable ectomycorrhizal forming species of the genera *Cortinarius*, *Hysterangium*, *Laccaria*, *Tricholoma* and *Phaeocollybia*, have also been mentioned from the páramo by Halling and Mueller (1999). Therefore, further mycorrhizal associations with other fungal species for *Com. arbutoides* can be assumed.

The genus *Cortinarius* is assumed to be the species-richest genus of Agaricales, containing over 2000 species (Garnica et al. 2005) with a worldwide distribution (Peintner et al. 2004). The taxonomy of *Cortinarius* is largely based on macromorphological characters, spore morphology as well as on chemical characters (Brandrud 1996). The subdivision of *Cortinarius* into subgeneric units causes many problems, induced by high morphological variation within species, as well as the different weighting of morphological characters by different taxonomists (Peintner et al. 2004). However, molecular investigation of the genus *Cortinarius* is just at the beginning (Liimatainen 2013; Zotti et al. 2014). As proposed by Peintner et al. (2004), studies should, first of all, focus on natural units (e.g. sections), bringing DNA sequence data as well as morphological and ecological data in accordance, as already done by several authors (e.g. Garnica et al. 2009, 2011; Suárez-Santiago et al. 2009; Niskanen et al. 2013a, b; Dima et al. 2014; Stensrud et al. 2014; Liimatainen et al. 2015).

*Cortinarius* is an important ectomycorrhizal fungal genus associated with trees, shrubs and a number of herbaceous plants of many different plant families (Liimatainen 2013), whereby also host specificity occurs (e.g. Brandrud 1996; Garnica et al. 2003; Frøslev et al. 2007; Niskanen et al. 2011; Liimatainen 2013). Based on fruit body collections, Halling and Mueller (1999; 2005), Mueller et al. (2006) and Ammirati et al. (2007) have reported and/or described 18 different Costa Rican Cortinarii. These species were collected in the Talamanca mountain range of Costa Rica, where *Comarostaphylis* and *Quercus* trees occur.

In our samples collected in the Cerro de la Muerte (Cordillera de Talamanca, Costa Rica) several different *Cortinarius* species formed mycorrhizas with *Com. arbutoides* and *Quercus* sp. The genus *Cortinarius* was identified using molecular methods such as large subunit (LSU) and internal transcribed spacer (ITS) sequencing as well as phylogenetic analysis. Plant primers were used to sequence the ITS region of the host plant from the same mycorrhizal system as used for fungal analysis. According to Agerer (1991), we present a morphological and anatomical description of four cortinarioid mycorrhizal systems associated with *Com. arbutoides*.

## Materials and methods

### Site location and sampling

Sampling was conducted in a secondary cloud forest around the Mountain Cerro de la Muerte (3491 m a.s.l.) in the Cordillera de Talamanca of Costa Rica, 54 km southeast of the capital city of San José. Site I (Estación Biologíca de la Muerte; 3100 m a.s.l.; 9° 33′ N, 83° 45′ W) is dominated by *Q. costaricensis* mixed with solitary individuals of *Com. arbutoides*. At site II (Reserva Forestal Los Santos; 3300 m a.s.l.; 9° 34′ N, 83° 45′ W), *Com. arbutoides* itself is the dominating species, mixed with a few isolated *Q. costaricensis*. Members of the Araliaceae (*Schefflera* and *Oreopanax*); Cunoniaceae (*Weinmannia*); Ericaceae (*Cavendishia*, *Disterigma* and *Vaccinium*); Poaceae (*Chusquea*); Primulaceae (*Myrsine*) and Winteraceae (*Drimys*) form the understory vegetation.

Fine root systems of *Com. arbutoides* were collected during the rainy seasons in October 2010 and 2011. For this, a soil corer (diameter 3 cm; length 40 cm) was used at distances of 50 and 100 cm from the trunk. At the University of Costa Rica, turgid and apparently healthy morphotypes were sorted out using a stereomicroscope. Systems with the same morphological features (e.g. colour, hydrophobicity presence, emanating elements and rhizomorphs) were assigned to one morphotype. For further analyses, the morphotypes were preserved in 2 % glutaraldehyde with a 0.1 M sodium cacodylate buffer (Münzenberger et al. 2009) for light microscopy or dried on silica gel for DNA extraction, respectively. Identification of each morphotype is based on their respective sequence type. Within these 2 years a total of 60 soil cores were taken and analysed. The genus *Cortinarius* was proven genetically in 23 soil cores.

### Molecular analyses

Genomic DNA was isolated from one unramified root tip per morphotype, using the DNeasy Plant Mini Kit (QIAGEN, Hilden, Germany) following the manufacturer’s recommendations. To identify the mycorrhizal fungi at both family and species level, PCR amplification and sequencing of the internal transcribed spacer (ITS) region and the ribosomal nuclear large subunit (LSU) were performed. Here, the primer combinations ITS1F/ITS4 (Gardes and Bruns 1993; White et al. 1990) as well as LR0R/LR5 (Moncalvo et al. 2000) were used. In order to identify the plant from mycorrhizal root tips without coamplifying fungal DNA the angiosperm-specific ITS primer pair ITS-5A/ITS-241r was amplified (Osmundson et al. 2007). Sequencing service was facilitated by GATC Biotech AG (Konstanz, Germany). A total of 399 root tips were analysed genetically, of which 33 were identified as members of the genus *Cortinarius*. All sequences were deposited in NCBI GenBank under the accession numbers KM456990-KM457022 (ITS), KM457023-KM457055 (LSU), KF419121 (*Com. arbutoides*) and KM978077 (*Quercus* sp.).

Sequences were analysed and edited using Chromas Lite v2.01 software (http://technelysium.com.au). Identity of obtained sequence data was confirmed by BLASTn search against the NCBI database (http://www.ncbi.nlm.nih.gov/) and the database UNITE (Kõljalg et al. 2005; http://unite.ut.ee/). For phylogenetic analysis at species level, the datasets of ITS sequences provided by Peintner et al. (2004), Garnica et al. (2005) and Ammirati et al. (2007) were used. The dataset was complemented by best match results obtained by NCBI and UNITE blast search for each sequence. Alignment was performed with MAFFT v7 (Katoh et al. 2002) using the FFT-NS-2 alignment algorithm. To estimate phylogenetic relationships, maximum likelihood and Bayesian approaches were applied. Maximum likelihood analyses were performed using RAxML (v7.7.1; Stamatakis 2006) in a parallelized version supplied by RAxML BlackBox (Stamatakis et al. 2008) with trees inferred from 100 rapid bootstrap analyses as starting trees in a heuristic search for the tree with the highest likelihood. GTRCAT was used in the heuristic search and the final evaluation of the best tree found was based on the GTR + Gamma model. The Bayesian analysis was performed using MrBayes v3.2.1 (Ronquist et al. 2012) on an iMac (2.9 GHz Quad-Core Intel Core i5). The GTR + Gamma model was in effect and four chains in two parallel runs were performed for 2,000,000 generations, sampling every 1000. Analyses were performed until average standard deviation of split frequencies was <0.01 and stationarity was checked using Tracer v1.6.1 (Rambaut et al. 2014). The first 50,000 trees were discarded before calculating the posterior probabilities. The potential scale reduction factor (PSRF) values for all inferences were ∼1.0, indicating a good posterior probability distribution sample.

### Microscopy

The morphological and anatomical description of the mycorrhizas was carried out according to Agerer (1987–2012; 1991), and the online key of DEEMY (Agerer and Rambold 2004–2015). Anatomical studies are based on multiple arbutoid mycorrhizal systems. Drawings were performed with an interference contrast microscope (BX50F-3, Olympus Corporation, Tokyo, Japan) connected with a drawing tube. All drawings were done in thousandfold magnification.

For semi-thin sections, the mycorrhizas were fixed with 2 % glutaraldehyde in 0.1 M sodium cacodylate buffer (pH 7.2) at room temperature until further processing. Thereafter, six washes in 0.1 M sodium cacodylate buffer were performed. Samples were postfixed in 1 % osmium tetroxide in the same buffer for 1 h under light exclusion at room temperature. After six washes in double-distilled water, samples were dehydrated by immersion for 15 min in 25, 50, 70 and 95 % acetone and three times for 1 h in 100 % acetone, respectively. The mycorrhizal tips were embedded in Spurr’s plastic (Spurr 1969) and sectioned with a diamond knife on an Ultracut Reichert Ultramicrotome (W. Reichert-LABTEC, Wolfratshausen, Germany). The sections (0.5-μm thick) were stained with crystal violet and investigated using a light microscope (Zeiss Axioskop 50, Oberkochen, Germany).

## Results

### Phylogenetic analysis

A total of 399 root tips were analysed genetically, of which 33 were assigned to the genus *Cortinarius* after sequence comparison with BLASTn in the NCBI database and UNITE. In NCBI, best matches were mainly received with samples originally from North America, whereas comparison in UNITE almost exclusively resulted in European species (Tab. 1). In NCBI, 11 samples (KKM 109, KKM 117, KKM 149, KKM 167, KKM 198, KKM 204, KKM 298, KKM 335, KKM 407, KKM 429, KKM 437) achieved their highest match with no further identified *Cortinarius* sp. or Cortinariaceae samples, whereby this was the case only for KKM 432 in UNITE. The highest identity match in NCBI (100 %) is obtained by KKM 144 with *Cortinarius comarostaphylii* from Costa Rica; in UNITE *C. leucophanes* from Finland is the closest match (99 %) for this sample. KKM 132 achieved the lowest identity match in both databases (89 as well as 88 %) with *Cortinarius exlugubris* from New Zealand and *Cortinarius terpsichores* from Sweden, respectively.

**Table 1:** Comparison of ITS sequences with NCBI and UNITE database obtained from 33 cortinarioid mycobionts associated with *Comarostaphylis arbutoides* or *Quercus* sp.

Samples	NCBI			Unite		
	Best match	Accession numbers	Identity in % (max. score)	Best match	Accession numbers	Identity in % (bit score)
KKM 81^I^	*Cortinarius* sp.	KM402900	98 (1129–1147)	*C. tillamookensis*	UDB015917	98 (1113–1131)
KKM 156^II^	*C. aurantiobasis*	DQ481866				
KKM 255^III^		DQ481899				
KKM 262^III^		FJ157139				
KKM 336^IV^		GQ159787				
KKM 344^IV^		HM068560				
KKM 359^IV^						
KKM 381^IV^						
KKM 388^IV^						
KKM 392^IV^						
KKM 431^IV^						
KKM 439^IV^						
KKM 109^II^	*Cortinarius* sp.	HQ285378	99 (1147–1179)	*C. fulvescens*	UDB018657	95–96 (1034–1047)
KKM 204^II^						
KKM 335^IV^						
KKM 437^IV^						
KKM 117^II^	*Cortinarius* sp.	EF619685	99 (1038–1040)	*C. ochrophyllus*	UDB000675	96 (921)
KKM 167^II^	Cortinariaceae	DQ377381				
KKM 132^II^	*C. exlugubris*	NR119791	89 (819)	*C. terpsichores*	UDB015909	88 (838)
KKM 144^II^	*C. comarostaphylii*	EF420151	100 (1216)	*C. leucophanes*	UDB019884	99 (1177)
		EF420153			UDB020289	
		NR131800				
	*C. oregonensis*	GQ159798				
KKM 149^II^	*Cortinarius* sp.	JQ711769	99 (1195)	*C. croceus*	UDB017892	99 (1162–1177)
KKM 429^IV^					UDB021432^a^	
KKM 177^II^	*C. tillamookensis*	KP087981	98 (1074)	*C. croceus*	UDB001554	98 (1061)
KKM 198^II^	*Cortinarius* sp.	GU998260	95 (887)	*C. casimiri*	UDB018229	95 (848)
		GU998441				
		GU998522				
KKM 298^III^	*Cortinarius* sp.	FJ196918	95 (883)	*C. anisatus*	UDB001317	93 (825)
KKM 330^III^	*C. camphoratus*	HQ604694	94 (850)	*C. raphanoides*	UDB018234	94 (874)
KKM 333^III^					UDB018300	
KKM 358^IV^	*C*. aff. *pauperculus*	GQ159858	98 (1125)	*C. fulvescens*	UDB018657	95 (1050)
KKM 373^IV^	*C. obtusus*	HQ604668	95 (1027)	*C. acutus*	UDB017978	93 (964)
KKM 376^IV^		HQ604670				
KKM 407^IV^	*Cortinarius* sp.	EF619685	99 (813)	*C*. cf. *bayeri*	UDB018640	99 (764)
	Cortinariaceae	DQ377381				
KKM 419^IV^	*Cortinarius* sp.	DQ481700	96 (941)	*C. alboviolaceus*	UDB018227	96 (913)
		FJ152517				
		KP403013				
	*C*. cf. *biformis*	HQ604700				
	*C. biformis*	FJ157106				
		FJ717531				
		FJ717532				
KKM 432^IV^	*C. cedriolens*	HQ604729	99 (1029)	*Cortinarius* sp.	UDB016059	98 (987)

Best match was chosen according to the highest maximum score or bit score. All showed matches obtained an *E* value of 0.0

Sites and year of sampling: site I in 2010 (^I^) and 2011 (^II^); site II in 2010 (^III^) and 2011 (^IV^)

^a^Sequence locked by author

Accessed 9 July 2015

**Fig. 1:**
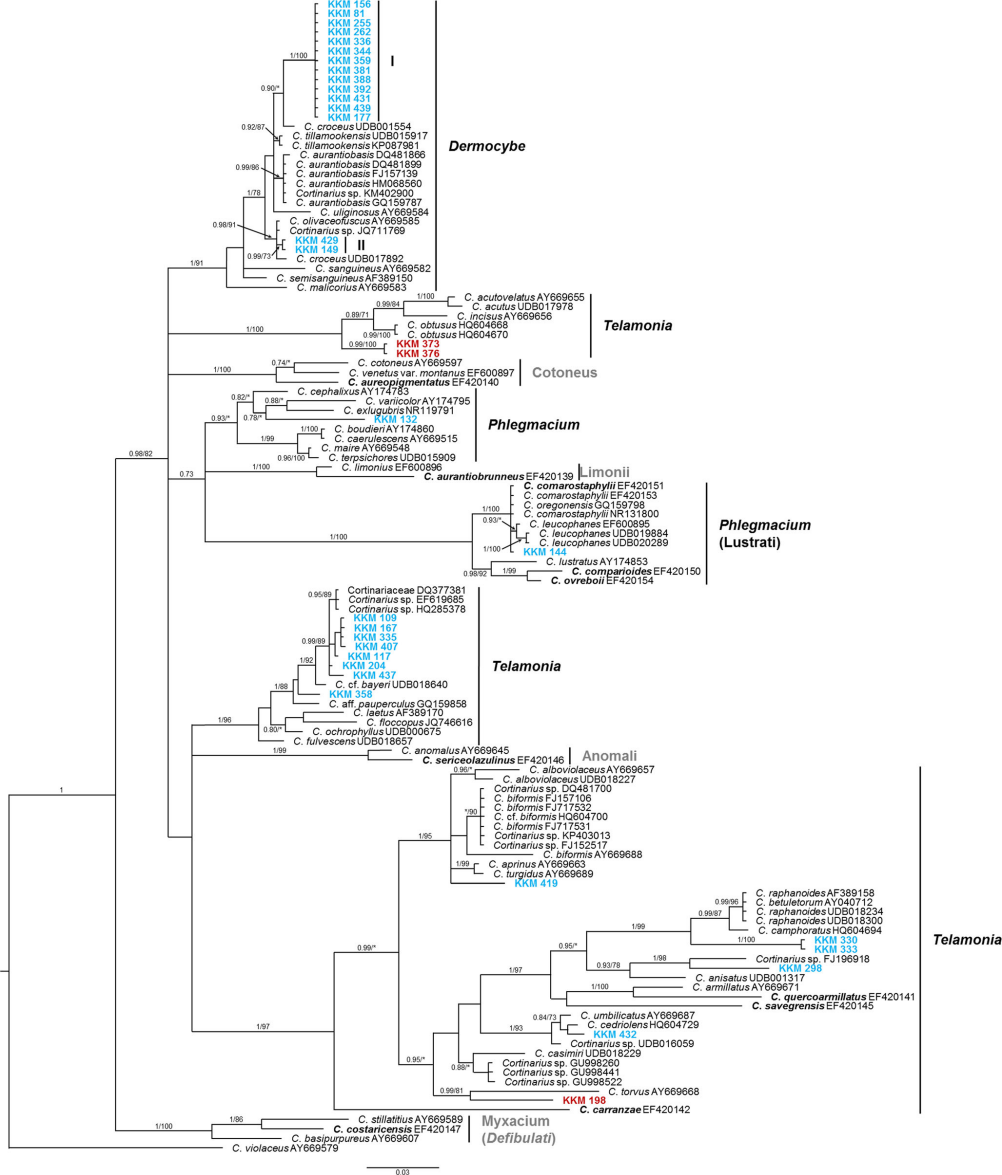
Phylogenetic relationship of 33 cortinarioid mycobionts associated with *Comarostaphylis arbutoides* (*blue*) or *Quercus* sp. (*red*) within selected representatives of the genus *Cortinarius*. Phylogram was obtained from Bayesian analysis based on ITS sequences. Branch support values were calculated as posterior probability from 2,000,000 generations of Bayesian analysis (*first number*), and as bootstrap support from RAxML analysis (*second number*). Values below 70 % are indicated with *asterisks* or omitted. The phylogram was rooted with *Cortinarius violaceus*. Costa Rican *Cortinarius* species from a previous study are marked in *bold* (Ammirati et al. 2007). Assignment to taxonomic units according to Peintner et al. (2004), Garnica et al. (2005), and Ammirati et al. (2007)

The Bayesian and RAxML phylogenies, generated by ITS sequences are concordant. Both trees show the same grouping structure, supported by mainly higher posterior probabilities (PP) in the Bayesian analysis and lower bootstraps (BS) in the RAxML analysis (Fig. 1). Within the genus *Cortinarius*, the 33 samples can be assigned to three different subgenera: *Dermocybe*, *Phlegmacium* and *Telamonia*.

Around half of the analysed cortinarioid sequences belong to the *Dermocybe* subgenus (PP 1/BS 91), whereas 13 samples are assigned to subgroup “Dermocybe I” (PP 1/BS 100), and a further two samples (KKM 149, KKM 429) to subgroup “Dermocybe II” (PP 0.99/BS 73) (Fig. 1). The samples KKM 132 and KKM 144 are assigned to subgenus *Phlegmacium*, but can be found in two different clades. There, KKM 144 is grouped in a very well-supported cluster (PP 1/BS 100), whereas the cluster with KKM 132 received a good PP (0.93), but a weak BS support (53). The remaining 16 cortinarioid samples are assigned to the subgenus *Telamonia* and are distributed amongst three very well-supported clusters (PP 1/BS 96 to 100) as shown in Fig. 1.

Except for the telamonioid samples KKM 198, KKM 373 and KKM 376, where *Quercus* sp. was proven as host plant (Fig. 1), all mycorrhizal systems of the cortinarioid samples are formed with *Com. arbutoides*.

### Morpho-anatomical descriptions of arbutoid mycorrhizas formed by various *Cortinarius* sp. species with the Ericaceae *Comarostaphylis arbutoides*

Of the 33 sequence types, four morphotypes were described in detail as, here, sufficient mycorrhizal materials were available. Assignment to the respective subgenus is based on phylogenetic analysis (Fig. 1).

Identification key for the cortinarioid mycorrhizas:

- **1** Mycorrhizal system usually not silvery, but densely stringy; older parts of mantle not transparent; rhizomorphs abundant, with hairy or even fan-like margins: **KKM 132 (*Phlegmacium*)**
- 1* Mycorrhizal system silvery, rapidly displaced by water when touched, then mantle transparent; epidermal cells generally visible through mantle along the whole mycorrhizal system; rhizomorphs frequent, with smooth margins: **2**
- **2** Mycorrhizal system densely silvery; all mantle layers with open anastomoses; no colour reaction with KOH and NH_4_OH: **KKM 298 (*Telamonia*)**
- 2* Mycorrhizal system slightly silvery; only inner mantle layer with open anastomoses; colour reaction with KOH and NH_4_OH: **3**
- **3** Mycorrhizal system ochre to yellowish brown, very tip yellowish; rhizomorphs ochre to yellowish brown; anastomoses of emanating elements likewise closed by a clamp or open: **KKM 255; KKM 359; KKM 388 (*Dermocybe*)**
- 3* Mycorrhizal system brown, very tip brownish to greyish; rhizomorphs ochre to reddish brown; anastomoses of emanating elements closed by a clamp or rarely open: **KKM 149 (*Dermocybe*)**

### KKM 132 (*Phlegmacium*) + *Com. arbutoides*

#### Morphological characters

**Fig. 2:**
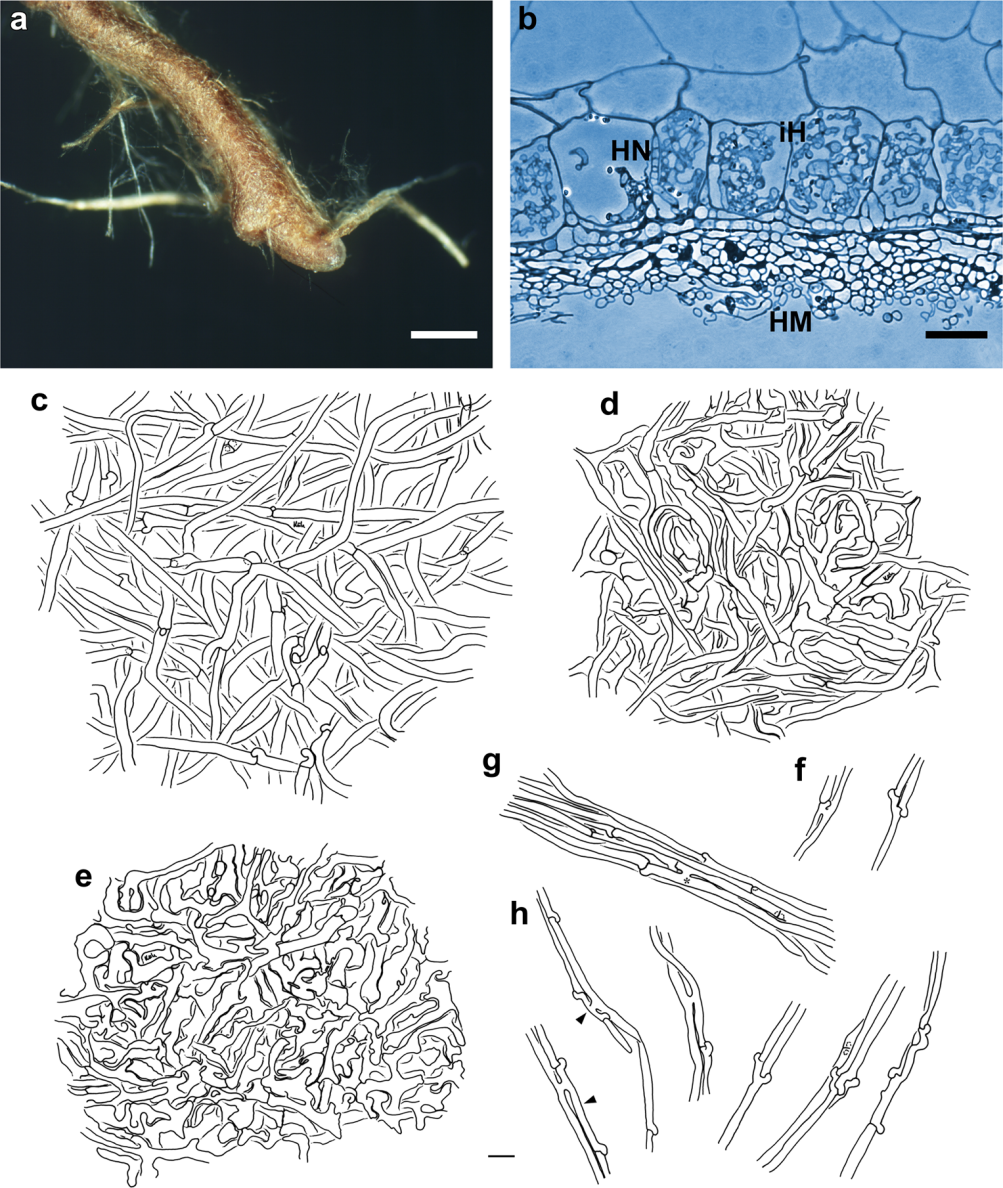
Arbutoid mycorrhiza of *Cortinarius* sp.—*Comarostaphylis arbutoides* (sample KKM 132, subgenus *Phlegmacium*). **a** Habit of the phlegmacioid mycorrhiza with ochre to brownish coloured mantle, densely stringy mantle surface and ochre to brownish rhizomorphs; *bar* = 0.5 mm. **b** Semi-thin section of phlegmacioid mycorrhiza with hyphal mantle (*HM*), Hartig net (*HN*) and intracellular hyphae (*iH*), *bar* = 20 μm. **c–h** Plan view of different mantle layers and emanating elements of the phlegmacioid mycorrhiza; *bar* = 10 μm. **c** Outer mantle layer with densely and irregularly arranged hyphae, hyphae with clamps. **d** Middle mantle layer with densely arranged hyphae, some hyphae in bundles, forming ring-like structures. **e** Inner mantle layer with densely arranged hyphae; hyphae irregular in shape. **f** Emanating hyphae with open anastomosis and backwards-oriented ramification. **g** Rhizomorph with open anastomosis (*asterisk*). **h** Hyphae of rhizomorphs with backwards-oriented clamps (*arrowheads*), open anastomoses and acute as well as backwards-oriented ramifications

(Fig. 2a) *Mycorrhizal systems* irregularly pinnate to dichotomous, with 0–1 orders of ramification, systems abundant and dense, up to 6.8 mm long, strongly hydrophobic, of medium distance fringe exploration type. *Main axes* 0.3–0.5 mm diameter. *Unramified ends* sinuous to tortuous, not inflated, cylindric, up to 1.1 (2.1) mm long and 0.2–0.3 mm diameter; mantle ochre to brownish, very tip ochre to yellowish, older parts dark brown and mycorrhizas not carbonising. *Surface of unramified ends* densely stringy, not smooth, very tip transparent and epidermal cells visible through mantle, older parts of mantle not transparent and occasionally, very tip partly silvery due to enclosed air. *Rhizomorphs* abundant, up to 0.12 mm diameter, roundish to flat in cross-section, emanating from all parts of the mycorrhiza, connection oblique, distal rhizomorphs connected over a long distance with mantle surface, growing into soil or organic layers, ochre to brownish, repeatedly ramified into smaller filaments, with hairy or even fan-like margins; rhizomorphs appear very wiry to brittle and are frequently found in short broken fragments around the mycorrhizal systems. *Cystidia* lacking. *Sclerotia* not observed.

#### Anatomical characters of the mantle in plan views

(Fig. 2c–e) Mantle lacks cells densely filled with oily droplets or brownish content, blue granules, needle-like contents, matrix, crystals and exudated pigments, as well as cystidia. *Outer mantle layers* densely plectenchymatous, hyphae irregularly to somewhat star-like arranged, rarely ramified, some hyphae in bundles, without any special pattern (type B, Agerer 1991) and yellow; hyphae (17) 25–39 (57) μm long, 2.7–4.4 μm in diameter, cells walls 0.3 (0.4) μm thick; hyphae with clamps and constricted at septa, septa as thick as cell walls. *Middle mantle layers* densely plectenchymatous, hyphae in bundles, forming ring-like structures, hyphae 7–39 (50) μm long, 1.6–4.6 (6.4) μm in diameter, cell walls (0.2) 0.3–0.4 μm thick, smooth and yellow; hyphae rarely septate, clampless and constricted at septa, septa as thick as cell walls. *Inner mantle layers* densely plectenchymatous, hyphae irregular in shape, hyphae (4) 8–27 (63) μm long, 1.8–6.8 (8.3) μm in diameter, cell walls 0.3–0.4 μm thick, smooth and yellow; septa not observed. *Very tip* like other parts of the mantle.

#### Anatomical characters of emanating elements

(Fig. 2f–h) Lacking are gelatinized hyphae, matrix, rhizomorphal nodia, simple septa, intrahyphal hyphae, crystals, brownish substances and secreted pigments; elbow-like protrusions not observed. *Rhizomorphs* undifferentiated, hyphae loosely interwoven and of uniform diameter (type A/B, Agerer and Rambold 2004–2015); hyphae smooth, cells (10) 65–113 μm long, 2.4–3.8 μm diameter and cell walls 0.3–0.4 μm; ramification backwards-oriented or acute, one or two hyphal diameter below the septum and ramifications one side branch at septum; septa with clamps, constricted at septa, backwards-oriented clamps observed only twice and septa as thick as hyphal walls; anastomoses frequent and open with a short bridge, bridge thinner or as thick as hyphae, cell walls of anastomoses as thick as remaining walls. *Emanating hyphae* straight to wavy, smooth, surface occasionally with few soil particles, cells 30–120 μm long, 2.0–3.4 (4.3) μm diameter, cell walls 0.3 μm, and distal ends of hyphae simple; ramification backwards-oriented or acute, with one side branch at septum, one or two hyphal diameter below the septum; septa with clamps, constricted at septa, backwards-oriented clamps not observed and septa as thick as hyphal walls; anastomoses rare and open with a short bridge, bridge as thick as hyphae, cell walls of anastomoses as thick as remaining walls. *Cystidia* not found.

#### Anatomical characters of longitudinal section

(Fig. 2b) Mantle plectenchymatous, 13–27 μm thick. Mantle of very tip plectenchymatous, 16–20 μm thick. Epidermal layer with intracellular hyphae and epidermal cells radially oval to eliptic; Hartig net around epidermal cells para-epidermal in one row and hyphal cells roundish to cylindrical. Tannin cells are lacking.

#### Colour reactions with different reagents (mantle preparations and emanating elements)

Cotton blue: hyphae blue or greenish; toluidine blue: hyphae blue to violet. No reaction was observed with: acetic acid, ethanol 70 %, Fe(II)SO_4_, guaiac, KOH 10 %, lactic acid, Lugol’s solution, Melzer’s reagent, NH_4_OH conc., sulpho-vanillin, H_2_SO_4_.

#### Reference specimen

Costa Rica, province of San José, canton of Pérez Zeledón, at mountain Cerro de la Muerte, Reserva Forestal Los Santos (3300 m a.s.l.; precipitation c. 2812 mm/year; inceptisol (USDA)), in a secondary cloud forest with *Q. costaricensis*, soil core exc., myc. isol. Katja Kühdorf; KKM 132, 12 October 2010; mycorrhiza deposited by B. Münzenberger (ZALF Müncheberg, Germany).

### KKM 298 (*Telamonia*) + *Com. arbutoides*

#### Morphological characters

**Fig. 3:**
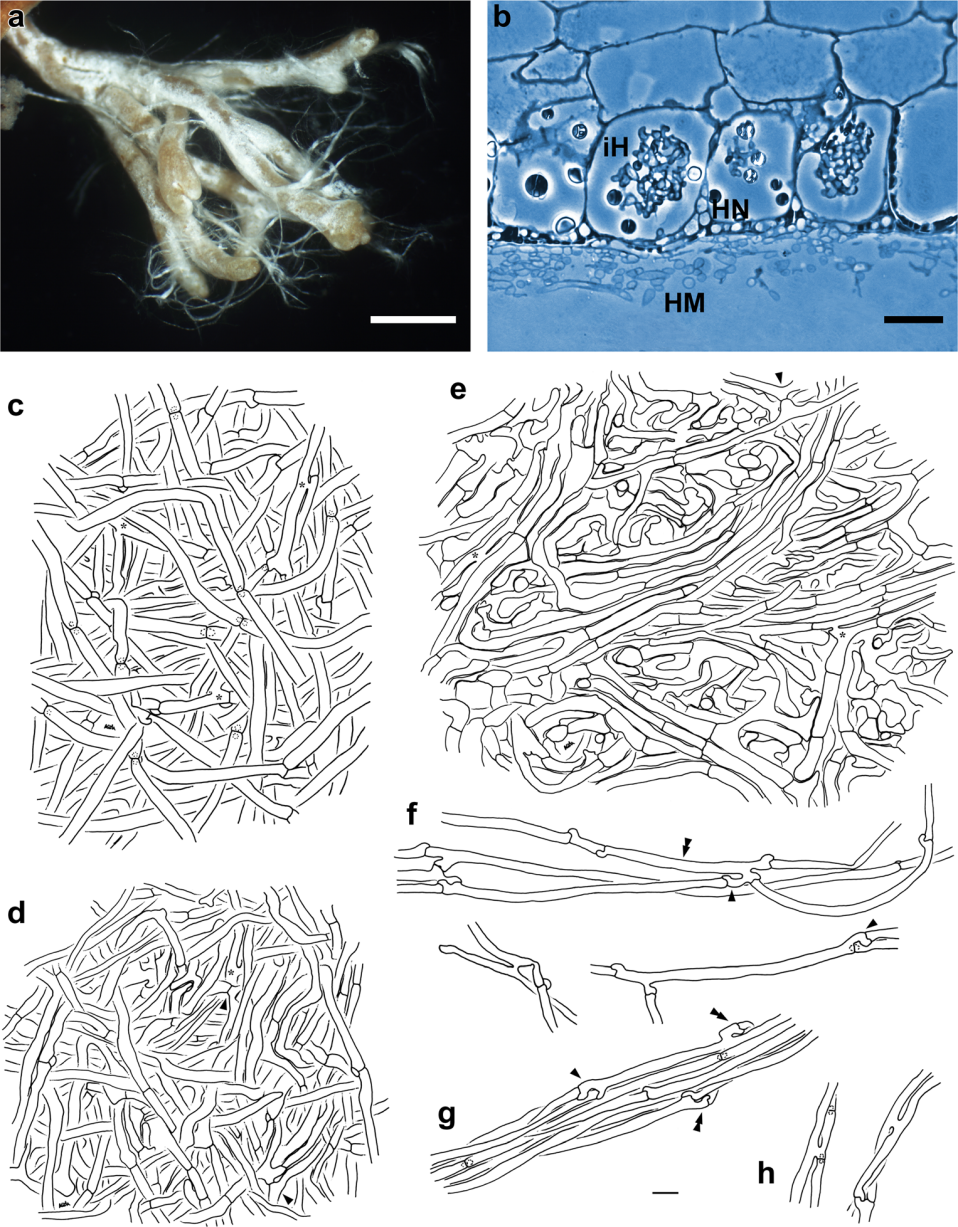
Arbutoid mycorrhiza of *Cortinarius* sp.—*Comarostaphylis arbutoides* (sample KKM 298, subgenus *Telamonia*). **a** Habit of the telamonioid mycorrhiza with transparent, yellowish mantle, partly densely stringy mantle surface, and white rhizomorphs; *bar =* 1 mm. **b** Semi-thin section of telamonioid mycorrhiza with hyphal mantle (*HM*), Hartig net (*HN*) and intracellular hyphae (*iH*); *bar =* 20 μm. **c–h** Plan view of different mantle layers and emanating elements of the telamonioid mycorrhiza; *bar =* 10 μm. **c** Outer mantle layer with densely and irregularly arranged hyphae; hyphae with clamps and open anastomoses (*asterisks*). **d** Middle mantle layer with densely arranged hyphae, some hyphae in bundles; hyphae with open (*asterisk*) or closed anastomoses (*arrowheads*). **e** Inner mantle layer with densely arranged hyphae, hyphae in bundles, forming ring-like structures; hyphae with open (*asterisks*) or closed (*arrowhead*) anastomoses. **f** Emanating hyphae with open anastomoses, backwards-oriented ramifications (*single arrowheads*) and backwards-oriented clamps (*double arrowhead*). **g** Rhizomorph with acute (*single arrowhead*) as well as backwards-oriented (*double arrowheads*) ramifications. **h** Hyphae of rhizomorphs with open anastomoses

(Fig. 3a) *Mycorrhizal systems* irregularly pinnate to dichotomous, with 0–4 orders of ramification, systems nest-like, forming hyphal mats, up to 8.4 mm long, strongly hydrophobic, of medium distance fringe exploration type. *Main axes* 0.3–0.4 mm diameter. *Unramified ends* bent or sinuous, not inflated, cylindric, up to 1.8 (3) mm long, 0.2–0.3 (0.4) mm diameter; mantle and very tip white and yellowish to ochre, older parts light orange, not carbonising. *Surface of unramified ends* densely stringy or forming rings, not smooth, fan-like cottony between side branches and main axis, densely silvery by enclosed air, rapidly displaced by water when touched, then mantle transparent and epidermal cells visible through mantle. *Rhizomorphs* frequent, up to 0.25 mm diameter; flat in cross-section, emanating from all parts of the mycorrhiza, connection oblique, distal rhizomorphs connected over a long distance with mantle surface, growing into soil or organic layers, white, repeatedly ramified into smaller filaments, with smooth margins. *Cystidia* lacking. *Sclerotia* not observed.

#### Anatomical characters of the mantle in plan views

(Fig. 3c–e) Mantle lacks cells densely filled with oily droplets or brownish content, blue granules, needle-like contents, crystals and exudated pigments, matrix, as well as cystidia. *Outer mantle layers* densely plectenchymatous, hyphae irregularly to somewhat star-like arranged, occasionally ramified, without any special pattern, often with bundles of parallel hyphae (type B, Agerer 1991) and colourless, with few soil particles; hyphae smooth and cylindric, hyphae (32) 85–140 μm long, (2.7) 3.3–5.8 (6.6) μm diameter, cell walls 0.2–0.3 μm thick; hyphae with clamps, constricted at septa, septa as thick as cell walls and anastomoses open, with a short bridge, bridge thinner or as thick as hyphae. *Middle mantle layers* densely plectenchymatous, hyphae irregularly interwoven, some hyphae in bundles, hyphae (14) 20–32 (50) μm long, (1.9) 2.3–4.3 (5.2) μm in diameter, cell walls 0.3 μm thick, irregularly inflated, smooth and colourless; hyphae with simple septa, occasionally with clamps, constricted at septa and septa as thick as cell walls and anastomoses open or closed, with short bridge, bridge thinner or as thick as hyphae. *Inner mantle layers* densely plectenchymatous, hyphae in bundles, forming ring-like structures, hyphae uneven in diameter, some hyphae epidermoid, sometimes ampullate at one side of septum and hyphae up to (9) 20–115 (200) μm long, 2.4–5.3 μm in diameter, cell walls 0.3 μm thick and colourless; hyphae with simple septa, rarely with clamp connection, constricted at septa, septa as thick as cell walls and anastomoses open, with short bridge, bridge thinner or as thick as hyphae, anastomoses closed, with long bridge, bridge bigger than hyphae. *Very tip* like other parts of the mantle.

#### Anatomical characters of emanating elements

(Fig. 3f–h) Lacking are gelatinized hyphae, matrix, rhizomorphal nodia, simple septa, intrahyphal hyphae, crystals, brownish substances and secreted pigments; elbow-like protrusions not observed . *Rhizomorphs* undifferentiated, hyphae loosely interwoven and of uniform diameter (type A/B, Agerer and Rambold 2004–2015); hyphae smooth, cells 60–100 μm long, 2.8–5.2 μm diameter and cell walls 0.3 μm; ramification backwards-oriented or acute, one or two hyphal diameter below the septum or in considerable distance from the septum ramification and ramifications one side branch at septum; septa with clamps, backwards-oriented clamps not observed and septa as thick as hyphal walls; anastomoses are frequent, open with a short bridge, bridge slightly thicker than hyphae and cell walls of anastomoses as thick as remaining walls; surface of peripheral hyphae with few soil particles. *Emanating hyphae* straight to wavy, smooth, surface occasionally with few soil particles, cells (25) 65–115 μm long, 2.4–4.8 μm diameter, cell walls 0.3 μm and distal ends of hyphae simple; ramification acute or approximately 90 °, with one side branch at septum and one or two hyphal diameter below the septum; septa with clamps, constricted at septa, backwards-oriented clamp observed only once, septa as thick as hyphal walls; anastomoses frequent, open with a short bridge, bridge thinner or as thick as hyphae and cell walls of anastomoses as thick as remaining walls. *Cystidia* not found.

#### Anatomical characters of longitudinal section

(Fig. 3b) Mantle plectenchymatous, 10–22 μm thick. Mantle of very tip plectenchymatous, 10–17 μm thick. Epidermal layer with intracellular hyphae and epidermal cells radially oval to eliptic; Hartig net around epidermal cells para-epidermal in one row and hyphal cells roundish to cylindrical. Tannin cells are lacking.

#### Colour reactions with different reagents (mantle preparations and emanating elements)

Cotton blue: hyphae blue; toluidine blue: hyphae violet and cell content pink. No reaction was observed with: acetic acid, ethanol 70 %, Fe(II)SO_4_, guaiac, KOH 10 %, lactic acid, Lugol’s solution, Melzer’s reagent, NH_4_OH conc., sulpho-vanillin, H_2_SO_4_.

#### Reference specimen

Costa Rica, province of San José, canton of Pérez Zeledón, at mountain Cerro de la Muerte, Estación Biológica de la Muerte (3100 m a.s.l.; precipitation c. 2812 mm/year; lithosol (FAO)), in a secondary cloud forest with *Q. costaricensis*, soil core exc., myc. isol. Katja Kühdorf; KKM 298, 4 October 2011; mycorrhiza deposited by B. Münzenberger (ZALF Müncheberg, Germany).

### KKM 255, KKM 359 and KKM 388 (*Dermocybe*) + *Com. arbutoides*

#### Morphological characters

**Fig. 4:**
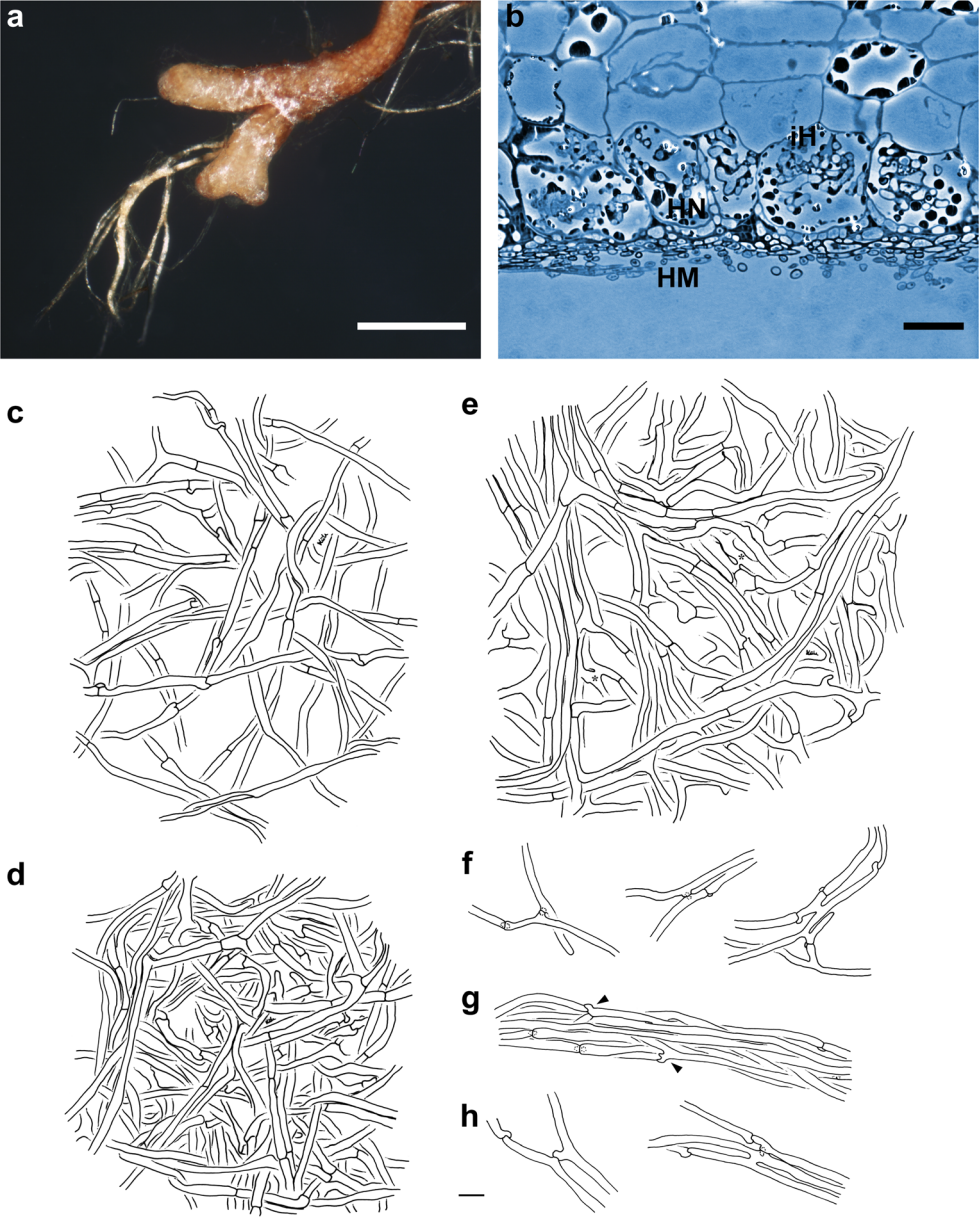
Arbutoid mycorrhiza of *Cortinarius* sp.—*Comarostaphylis arbutoides* (samples KKM 255, KKM 359 and KKM 388; subgenus *Dermocybe*). **a** Habit of the dermocyboid mycorrhiza with transparent, ochre to yellowish brown mantle, loosely stringy mantle surface and ochre to yellowish brown rhizomorphs; *bar =* 0.5 mm. **b** Semi-thin section of dermocyboid mycorrhiza with hyphal mantle (*HM*), Hartig net (*HN*) and intracellular hyphae (*iH*); *bar =* 20 μm. **c–h** Plan view of different mantle layers and emanating elements of the dermocyboid mycorrhiza; *bar =* 10 μm. **c** Outer mantle layer with loosely and irregularly arranged hyphae, some hyphae in bundles and hyphae with clamps. **d** Middle mantle layer with densely arranged hyphae, some hyphae in bundles. **e** Inner mantle layer with densely arranged hyphae, hyphae in bundles, forming ring-like structures; hyphae with open anastomoses (*asterisks*). **f** Emanating hyphae with contact clamps and open anastomoses. **g** Rhizomorph with acute ramifications (*arrowheads*). **h** Hyphae of rhizomorphs with contact clamps and open anastomosis

(Fig. 4a) *Mycorrhizal systems* irregularly pinnate to dichotomous, with 0–2 orders of ramification, systems solitary or in small numbers to abundant and dense, up to 9.9 mm long, slightly hydrophobic, of medium distance fringe exploration type. *Main axes* 0.2–0.6 mm diameter. *Unramified ends* bent or sinuous, not inflated, cylindric, up to 1.5 (2.7) mm long, 0.2–0.3 mm diameter; mantle ochre to yellowish brown, very tip yellowish, older parts dark orange and mycorrhizas not carbonising. *Surface of unramified ends* loosely stringy to loosely cottony, not smooth, between side branches and main axis sometimes fan-like cottony, slightly silvery by enclosed air, rapidly displaced by water when touched, then mantle generally transparent; epidermal cells visible through mantle. *Rhizomorphs* frequent, up to 0.11 mm diameter; flat in cross-section, emanating from all parts of the mycorrhiza, connection oblique, distal rhizomorphs connected over a long distance with mantle surface, growing into soil or organic layers, ochre to yellowish brown, repeatedly ramified into smaller filaments, with smooth margins. *Cystidia* lacking. *Sclerotia* not observed.

#### Anatomical characters of the mantle in plan views

(Fig. 4c–e) Mantle lacks cells densely filled with oily droplets or brownish content, blue granules, needle-like contents, matrix, crystals and exudated pigments, as well as cystidia. *Outer mantle layers* loosely plectenchymatous, hyphae irregularly arranged, some hyphae in bundles, rarely ramified, without any special pattern (type B, Agerer 1991), colourless and with few soil particles; hyphae 25–90 μm long, 1.6–4.2 μm in diameter and cells walls 0.3 μm thick and hyphae with clamps, constricted at septa, septa as thick as cell walls. *Middle mantle layers* densely plectenchymatous, hyphae irregularly interwoven, some hyphae in bundles, occasionally ampullate at one side of septum, hyphae 15–50 μm long, 2.2–3.8 μm in diameter, cell walls 0.3 μm thick, smooth and colourless; hyphae with simple septa, rarely with clamps and constricted at septa, septa as thick as cell walls. *Inner mantle layers* densely plectenchymatous, hyphae in bundles, forming ring-like structures, occasionally ampullate at one side of septum, hyphae 17–200 μm long, 1.9–4.6 (5.3) μm in diameter, cell walls 0.3 (0.4) μm thick, smooth and colourless; hyphae with simple septa, constricted at septa and septa as thick as cell walls; anastomoses open, with short bridge, bridge thinner or as thick as hyphae. *Very tip* like other parts of the mantle.

#### Anatomical characters of emanating elements

(Fig. 4f–h) Lacking are gelatinized hyphae, matrix, rhizomorphal nodia, simple septa, intrahyphal hyphae, crystals, brownish substances and secreted pigments; elbow-like protrusions are not observed. *Rhizomorphs* undifferentiated, hyphae loosely interwoven and of uniform diameter (type A/B, Agerer and Rambold 2004–2015); hyphae smooth, cells 30–125 μm long, 2.8–4.3 μm diameter and cell walls 0.3 μm; ramification acute, one or two hyphal diameter below the septum and ramifications one side branch at septum; septa with clamps, constricted at septa, septa as thick as hyphal walls; anastomoses frequent, open or closed by a clamp (contact clamp), with a short bridge or bridge almost lacking, bridge as thick as hyphae, cell walls of anastomoses as thick as remaining walls. *Emanating hyphae* straight to wavy, smooth, surface occasionally with few soil particles, cells 40–110 μm long, 2.1–3.8 μm diameter, cell walls 0.3 μm and distal ends of hyphae simple; ramification acute or approximately 90 °, with one side branch at septum and one or two hyphal diameter below the septum; septa with clamps, constricted at septa, septa as thick as hyphal walls; anastomoses are frequent, open or closed by a clamp (contact clamp), with a short bridge or bridge almost lacking, bridge as thick as hyphae, cell walls of anastomoses as thick as remaining walls. *Cystidia* not found.

#### Anatomical characters of longitudinal section

(Fig. 4b) Mantle plectenchymatous, 5–19 μm thick. Mantle of very tip plectenchymatous, 4–14 μm thick. Epidermal layer with intracellular hyphae, epidermal cells radially oval to eliptic; Hartig net around epidermal cells para-epidermal in one row and hyphal cells roundish to cylindrical. Tannin cells are lacking.

#### Colour reactions with different reagents (mantle preparations and emanating elements)

Cotton blue: hyphae blue to patchy greenish; KOH 10 %: rhizomorphs bright pink; NH_4_OH conc.: rhizomorphs bright pink after a few minutes; toluidine blue: hyphae violet, cell content pink. No reaction was observed with: acetic acid, ethanol 70 %, Fe(II)SO_4_, guaiac, lactic acid, Lugol’s solution, Melzer’s reagent, sulpho-vanillin, H_2_SO_4_.

#### Reference specimen

Costa Rica, province of San José, canton of Pérez Zeledón, at mountain Cerro de la Muerte, Estación Biológica de la Muerte (3100 m a.s.l.; precipitation c. 2812 mm/year; lithosol (FAO)), in a secondary cloud forest with *Q. costaricensis*, soil core exc., myc. isol. Katja Kühdorf; KKM 255, 4 October 2011 and mycorrhiza deposited by B. Münzenberger (ZALF Müncheberg, Germany). *Further material studied* Costa Rica, province of San José, canton of Pérez Zeledón, at mountain Cerro de la Muerte, Reserva Forestal Los Santos (3300 m a.s.l.; precipitation c. 2812 mm/year; inceptisol (USDA)), in a secondary cloud forest with *Q. costaricensis*, soil core exc., myc. isol. Katja Kühdorf; KKM 359 and KKM 388, 18 October 2011; mycorrhiza deposited by B. Münzenberger (ZALF Müncheberg, Germany).

### KKM 149 (*Dermocybe*) + *Com. arbutoides*

#### Morphological characters

**Fig. 5:**
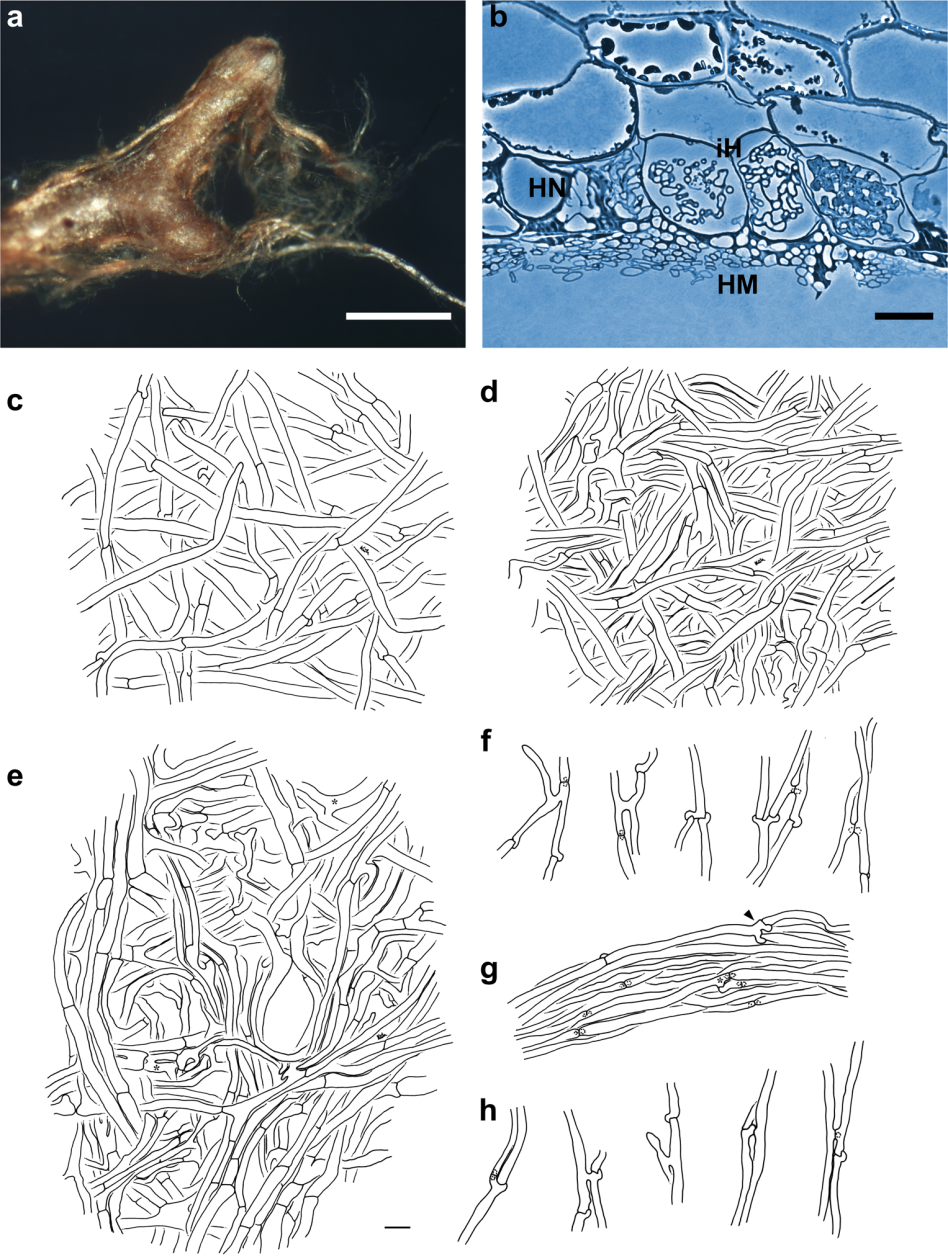
Arbutoid mycorrhiza of *Cortinarius* sp.—*Comarostaphylis arbutoides* (sample KKM 149, subgenus *Dermocybe*). **a** Habit of the dermocyboid mycorrhiza with transparent, brown-coloured mantle, loosely stringy mantle surface and ochre to reddish brown rhizomorphs = *bar* 0.5 mm. **b** Semi-thin section of dermocyboid mycorrhiza with hyphal mantle (*HM*), Hartig net (*HN*) and intracellular hyphae (*iH*); *bar* = 20 μm. **c–h** Plan view of different mantle layers and emanating elements of the dermocyboid mycorrhiza; *bar* = 10 μm. **c** Outer mantle layer with loosely and irregularly arranged hyphae; hyphae with clamps. **d** Middle mantle layer with densely arranged hyphae, some hyphae in bundles. **e** Inner mantle layer with densely arranged hyphae, hyphae in bundles, forming ring-like structures; hyphae with open anastomoses (*asterisks*). **f** Emanating hyphae with open anastomoses (*single arrowheads*), backwards-oriented ramification and with contact clamps. **g** Rhizomorph with contact clamp (*asterisk*) and acute ramification (*arrowhead*). **h** Hyphae of rhizomorphs with acute ramification, open anastomoses and contact clamps

(Fig. 5a) *Mycorrhizal systems* irregularly pinnate to dichotomous, with 0–2 orders of ramification, systems abundant and dense, up to 11.3 mm long, slightly hydrophobic and of medium distance fringe exploration type. *Main axes* 0.3–0.5 mm diameter. *Unramified ends* sinuous to tortuous, not inflated, cylindric, up to 1.6 (2.5) mm long, 0.3 mm diameter; mantle brown, very tip brownish to greyish, older parts dark brown and mycorrhizas not carbonising. *Surface of unramified ends* loosely stringy to loosely cottony, not smooth, between side branches and main axis sometimes fan-like cottony, slightly silvery by enclosed air, rapidly displaced by water when touched, then mantle generally transparent and epidermal cells visible through mantle. *Rhizomorphs* frequent, up to 0.13 mm diameter, flat in cross-section, emanating from all parts of the mycorrhiza, connection oblique, distal rhizomorphs connected over a long distance with mantle surface, growing into soil or organic layers, ochre to reddish brown, repeatedly ramified into smaller filaments, with smooth margins. *Cystidia* lacking. *Sclerotia* not observed.

#### Anatomical characters of the mantle in plan views

(Fig. 5c–e) Mantle lacks cells densely filled with oily droplets or brownish content, blue granules, needle-like contents, matrix, crystals and exudated pigments, as well as cystidia. *Outer mantle layers* loosely plectenchymatous, hyphae irregularly arranged, rarely ramified, without any special pattern (type B, Agerer 1991), colour yellowish to colourless and with few soil particles; hyphae 20–59 (100) μm long, 3.5–4.7 μm in diameter, cells walls 0.3 (0.4) μm thick; hyphae with clamps, constricted at septa and septa as thick as cell walls. *Middle mantle layers* densely plectenchymatous, some hyphae in bundles or irregularly interwoven, sometimes ampullate at one side of septum, hyphae 20–110 μm long, 2.8–5.8 μm in diameter, cell walls 0.3 μm thick, smooth and colourless; hyphae with simple septa, rarely with clamps, constricted at septa and septa as thick as cell walls. *Inner mantle layers* are densely plectenchymatous, hyphae in bundles, forming ring-like structures, sometimes ampullate at one side of septum, hyphae uneven in diameter, hyphae 30–200 μm long, 1.3–5.1 μm in diameter, cell walls 0.3–0.4 μm thick, smooth and colourless; hyphae with simple septa, constricted at septa and septa as thick as cell walls; anastomoses open with short bridge, bridge thinner or as thick as hyphae. *Very tip* is like other parts of the mantle.

#### Anatomical characters of emanating elements

(Fig. 5f–h) Lacking are gelatinized hyphae, matrix, rhizomorphal nodia, simple septa, intrahyphal hyphae, crystals, brownish substances and secreted pigments; elbow-like protrusions not observed. *Rhizomorphs* undifferentiated, hyphae loosely interwoven and of uniform diameter (type A/B, Agerer and Rambold 2004–2015); hyphae smooth, cells 20–90 μm long, 2.4–3.8 μm diameter, cell walls 0.3 μm; ramification acute, one or two hyphal diameter below the septum and ramifications one side branch at septum; septa with clamps, constricted at septa and septa as thick as hyphal walls; anastomoses are frequent, closed by a clamp or rarely open, with a short bridge or bridge almost lacking, bridge as thick as hyphae and cell walls of anastomoses as thick as remaining walls. *Emanating hyphae* straight to wavy, smooth, surface occasionally with few soil particles, cells 20–95 μm long, 2.1–3.8 (4.7) μm diameter, cell walls 0.3 μm, and distal ends of hyphae simple; ramification acute or approximately 90 °, with one side branch at septum, one or two hyphal diameter below the septum, backwards-oriented ramification observed only once; septa with clamps, constricted at septa and septa as thick as hyphal walls; anastomoses frequent, closed by a clamp (contact clamp) or rarely open, with a short bridge or bridge almost lacking, bridge as thick as hyphae and cell walls of anastomoses as thick as remaining walls. *Cystidia* not found.

#### Anatomical characters of longitudinal section

(Fig. 5b) Mantle plectenchymatous, 4–16 μm thick. Mantle of very tip plectenchymatous, 4–11 μm thick. Epidermal layer with intracellular hyphae and epidermal cells radially oval to eliptic; Hartig net around epidermal cells para-epidermal in one row and hyphal cells roundish to cylindrical. Tannin cells are lacking.

#### Colour reactions with different reagents (mantle preparations and emanating elements)

Cotton blue: hyphae blue to patchy greenish; KOH 10 %: rhizomorphs bright pink; NH_4_OH conc.: rhizomorphs bright pink after a few minutes; toluidine blue: hyphae violet and cell content pink. No reaction was observed with: acetic acid, ethanol 70 %, Fe(II)SO_4_, guaiac, lactic acid, Lugol’s solution, Melzer’s reagent, sulpho-vanillin, H_2_SO_4_.

#### Reference specimen

Costa Rica, province of San José, canton of Pérez Zeledón, at mountain Cerro de la Muerte, Reserva Forestal Los Santos (3300 m a.s.l.; precipitation c. 2812 mm/year; inceptisol (USDA)), in a secondary cloud forest with *Q. costaricensis*, soil core exc., myc. isol. Katja Kühdorf; KKM 149, 12 October 2010; mycorrhiza deposited by B. Münzenberger (ZALF Müncheberg, Germany).

## Discussion

Despite the efforts in investigating the biodiversity of Costa Rican Cortinarii, only Ammirati et al. (2007) supplied genetic data for their reported species. This makes it difficult to address mycorrhizal findings to already known species. With one exception, none of the cortinarioid mycorrhizas, found in Costa Rica, can be determined to species level.

However, due to sequence identity of 100 %, the mycorrhizal sample KKM 144 can most probably be assigned to *C. comarostaphylii*. This species occurs in the Costa Rican páramo (Halling and Mueller 1999) and, according to Ammirati et al. (2007), *C. comarostaphylii* seems to be restricted to the Ericaceae *Com. arbutoides*. This plant was also confirmed genetically as host plant for the mycorrhiza KKM 144. Due to strict host specificity of many *Cortinarius* species (e.g. Brandrud 1996; Liimatainen 2013), identification with the European species *C. leucophanes* (Fig. 1) is excluded, since this species is associated with conifers (Ammirati et al. 2007).

Based on phylogenetic analyses, the sample KKM 132 is placed in the subgenus *Phlegmacium*, but fitted laboriously into this subgenus during processing. Also, the databases NCBI and UNITE reached comparatively low identity values, which confirms the insufficient sequence data quantity of Costa Rican Cortinarii. However, beside *Cortinarius glaucopus*, Halling and Mueller (1999) mention another phlegmacioid Cortinarii from the Costa Rican páramo. Since no sequence data is available, it remains unclear, whether or not this unidentified *Phlegmacium* species correspond to our mycorrhizal sample KKM 132.

### Mycorrhiza assigned to subgenus *Phlegmacium*

The mycorrhiza KKM 132 strongly differs in morphology from phlegmacioid ectomycorrhizas (ECMs) hitherto described in DEEMY (*Cortinarius alnobetulae* (Wiedmer and Senn-Irlet 1999a), *Cortinarius calochrous* ssp. *coniferarum* (Kernaghan 2001), *Cortinarius kuehneri* (Wiedmer and Senn-Irlet 1999b), *C. odorifer* (Uhl 1988; Egli 1992), as well as *Cortinarius variecolor* (Agerer 1969, 1988)). All these ECMs offer a silvery surface, whereas our *Phlegmacium* sample from Costa Rica lacks this feature. However, amongst this feature and its dense stringy surface, the ochre to yellowish colour, as well as the lack of sclerotia, KKM 132 resembles the Cortinariaceae “*Nothofagirhiza reticulosa*” from Chile (Palfner 2001; Agerer and Rambold 2004–2015).

Anatomically, KKM 132 resembles “*N. reticulosa*” in showing similar plectenchymatous mantle structures. However, in contrast to KKM 132, “*N. reticulosa*” possess anastomoses in the outer mantle layer, a rough hyphal surface in the outer mantle layer as well as in the emanating elements, and simple septa as well as intrahyphal hyphae in the emanating hyphae.

The phlegmacioid mycorrhiza of KKM 132 shows no reaction with KOH, an attribute in common with *C. alnobetulae* and “*N. reticulosa*”. However, both samples differ from KKM 132 in lacking reaction with cotton blue and toluidine blue, and a positive reaction with sulpho-vanillin, respectively. The likewise absent reaction with NH_4_OH of KKM 132 was not investigated in the other mycorrhizal samples of *Phlegmacium*.

### Mycorrhiza assigned to subgenus *Telamonia*

In DEEMY and literature, the following ECMs of the subgenus *Telamonia* are described as follows: *Cortinarius armillatus* (Cuvelier 1990; Cuvelier and Agerer 1991), *Cortinarius atropusillus* (Wiedmer and Senn-Irlet 1999c), *Cortinarius badiovestitus* (Wiedmer and Senn-Irlet 1999d), *Cortinarius bibulus* (Wiedmer and Senn-Irlet 2001a), *Cortinarius bulliardii* (Raidl et al. 2006), *Cortinarius cinnabarinus* (Ceruti et al. 1988; Brand 1991, 1992), *Cortinarius helvelloides* (Wiedmer and Senn-Irlet 2001b), *Cortinarius hinnuleus* (Kovács et al. 2002), *Cortinarius malachius* (Uhl 1988), *Cortinarius obtusus* (Agerer 1987a; Gronbach 1988; Gronbach and Agerer 1988) and *Cortinarius saturninus* (Seress et al. 2012). Here, *C. obtusus* resembles our telamonioid mycorrhiza KKM 298 with the silvery surface, white rhizomorphs with smooth margins, and the medium distance fringe exploration type.

Anatomically, the KKM 298 differs clearly from the 11 *Cortinarius* subgenus *Telamonia* ECMs. *C. badiovestitus*, *C. bibulous*, *C. hinnuleus* and *C. obtusus* have contact clamps in their emanating elements, whereas KKM 298 possesses no contact clamps at all. The anastomoses of *C. cinnabarinus* are not only open with a short bridge but are also closed by a clamp with a long bridge. Moreover, together with *C. bulliardii* and *C. malachius* and *C. cinnabarinus* show differentiated rhizomorphs of type D or C, whereas these of KKM 298 are assigned to type A/B. The mycorrhiza of *C. saturninus* is the only one which offers a matrix in the mantle layers as well as in the rhizomorphs. Together with *C. armillatus*, both systems feature not only clamps but also secondary septa in the emanating elements which were, in contrast, not observed in KKM 298. None of the *Cortinarius* species possess open anastomoses in all three mantle layers, whereby, in the case of *C. cinnabarinus*, *C. hinnuleus* and *C. saturninus* no middle mantle layer was described. Secondly, a distinctly ring-like arrangement of the inner mantle layer, as was also shown for KKM 298, was not described in any of the 11 telamonioid *Cortinarius* ECMs.

For chemical reactions, only KOH was tested in all described *Cortinarius* subgenus *Telamonia* ECMs. Here, no reaction was observed for KKM 298, as well as for most of the other telamonioid species. Exceptions are *C. bulliardii*, *C. cinnabarinus* and *C. malachius* which show violet pink, red-violet colour reactions or dissolving incrustations. A likewise negative reaction with NH_4_OH, as shown for KKM 298, cannot be conformed since the other telamoinioid systems were not tested. For *C. armillatus*, no information on chemical reactions is given at all.

### Mycorrhizas assigned to subgenus *Dermocybe*

Currently, nine ECMs of the subgenus *Dermocybe* are described in DEEMY: *Dermocybe cinnamomea* (Agerer 1987b; Agerer and Gronbach 1987; Gronbach 1988; Berg 1989; Weiss 1988), *Dermocybe cinnamomeolutea* (Waller and Agerer 1993), *Dermocybe crocea* (Uhl and Agerer 1987, 1988; Uhl 1988), *Dermocybe holoxantha* (Uhl 1988; Agerer 1995), *Dermocybe huronensis* (Kuss et al. 2004), *Dermocybe palustris* (Uhl and Agerer 1987; Agerer 1995), *Dermocybe phoenicea* (Cuvelier 1990), *Dermocybe sanguinea* (Agerer 1987b, c), as well as *Dermocybe semisanguinea* (Uhl 1988; Agerer and Uhl 1989; Agerer 1995). Due to the ochre to yellowish brown-coloured mantle and rhizomorphs, the mycorrhizal systems of KKM 255, KKM 359 and KKM 388 (“Dermocybe I”) are similar to *D. cinnamomeolutea*. In contrast, the system of KKM 149 (“Dermocybe II”) shows a brown to dark brown mantle with a reddish tint, as is also common with *D. phoenicea*. However, both described mycorrhizas show a distinctly transparent mantle clearly distinguishing them from the other *Dermocybe* species described in DEEMY so far.

Anatomically, the mycorrhizas of “Dermocybe I” and “Dermocybe II” do not differ much. Both types show a plectenchymatous mantle throughout undifferentiated rhizomorphs, infrequently with smooth margins (types A or B) and both form short anastomoses with contact clamps. These features are typical for *Dermocybe* species (Agerer 1995). According to DEEMY, our samples herein resemble *D. cinnamomeolutea*, *D. holoxantha* and *D. huronensis*. Nevertheless, these three species also show clear differences. Hyphae of emanating elements (*D. huronensis*), as well as of the outer mantle layer (*D. cinnamomeolutea*) possess a rough surface. *D. cinnamomeolutea* and *D. holoxantha* have a matrix in the middle and inner mantle layers and, furthermore, none of these three species show a ring-like inner mantle layer as occurs in both, “Dermocybe I” and “Dermocybe II”. Anastomoses open or closed by a clamp, are very common in the emanating elements, whereas in “Dermocybe II”, open anastomoses are less frequently observed than in “Dermocybe I”. Within the described *Dermocybe* ECM only, *D. phoenicea* also feature these two types of anastomoses.

The rhizomorphs of “Dermocybe I” and “Dermocybe II” show a bright pink colour reaction with KOH, as well as with NH_4_OH. A colour reaction with NH_4_OH was only tested in *D. cinnamomea*, where no reaction was observed. A reaction with KOH was investigated in seven of the nine *Dermocybe* species, where again, *D. cinnamomea*, as well as *D. palustris* showed a reaction, but which only affected the hyphal content or the mantle, respectively.

According to Ammirati et al. (2007), Costa Rica has a limited number of confirmed host plants for ectomycorrhizal fungi, such as *Quercus* sp., *Com. arbutoides* and *Alnus acuminate*. Even though oak species have an extensive distribution and are the most common ectomycorrhizal host plant for *Cortinarius*, they only occur in small numbers. Additionally, some *Cortinarius* species have very special habitat preferences (Liimatainen 2013) and, amongst topographical factors, often only exist in small, geographically isolated populations (Frøslev et al. 2005). Beside insufficient recorded biodiversity, this explains why the *Cortinarius* findings in this study primarily differ from Ammirati et al. (2007), and why species of, e.g. subgenus *Dermocybe* have not been reported yet for Costa Rica. Moreover, in contrast to Ammirati et al. (2007), who described Costa Rican Cortinarii sporocarps exclusively found in June, we found no fruit bodies at all. This suggests that sampling in October, within the rainy season, is suitable for mycorrhizal investigations but not for *Cortinarius* fruit bodies in this area.
